# Stage-Dependent Regulation of Dental Pulp Stem Cell Odontogenic Differentiation by Transforming Growth Factor-*β*1

**DOI:** 10.1155/2022/2361376

**Published:** 2022-10-26

**Authors:** Yu Bai, Xin Liu, Junqing Li, Zhihua Wang, Qian Guo, Min Xiao, Paul R. Cooper, Qing Yu, Wenxi He

**Affiliations:** ^1^State Key Laboratory of Military Stomatology & National Clinical Research Center for Oral Diseases & Shaanxi Key Laboratory of Stomatology, Department of Operative Dentistry and Endodontics, School of Stomatology, Air Force Medical University, 145 Changle Road, Xi'an 710032, China; ^2^Hospital of Stomatology, Zunyi Medical University, 89 Wu-jiang Dong Road, Zunyi 563003, China; ^3^Department of Oral Sciences, Sir John Walsh Research Institute, Faculty of Dentistry, University of Otago, P.O. Box 56, Dunedin 9054, New Zealand; ^4^Department of Stomatology, Air Force Medical Center, Air Force Medical University, 30 Fucheng Road, Beijing 100142, China

## Abstract

Transforming growth factor-*β*1 (TGF-*β*1) is an important multifunctional cytokine with dual effects on stem cell differentiation. However, the role of TGF-*β*1 on odontogenic differentiation of dental pulp stem cells (DPSCs) remains to be entirely elucidated. In the present study, we initially investigated the effect of TGF-*β*1 at a range of concentrations (0.1-5 ng/mL) on the proliferation, cell cycle, and apoptosis of DPSCs. Subsequently, to determine the effect of TGF-*β*1 on odontogenic differentiation, alkaline phosphatase (ALP) activity and Alizarin Red S (ARS) staining assays at different concentrations and time points were performed. Quantitative real-time polymerase chain reaction (qRT-PCR) and Western blot analysis were used to determine the levels of odonto-/osteo-genic differentiation-related gene and protein expression, respectively. For *in vivo* studies, newly formed tissue was assessed by Masson's trichrome and von Kossa staining. Data indicated that TGF-*β*1 inhibited DPSCs proliferation in a concentration-and time-dependent manner (*p* < 0.05) and induced cell cycle arrest but did not affect apoptosis. ALP activity was enhanced, while ARS reduced gradually with increasing TGF-*β*1 concentrations, accompanied by increased expression of early marker genes of odonto-/osteo-genic differentiation and decreased expression of late-stage mineralization marker genes (*p* < 0.05). ALP expression was elevated in the TGF-*β*1-treatment group until 14 days, and the intensity of ARS staining was attenuated at days 14 and 21 (*p* < 0.05). Compared with the control group, abundant collagen but no mineralized tissues were observed in the TGF-*β*1-treatment group *in vivo*. Overall, these findings indicate that TGF-*β*1 promotes odontogenic differentiation of DPSCs at early-stage while inhibiting later-stage mineralization processes.

## 1. Introduction

Progress in the development of novel biomaterials and advances in our understanding of pulp biology, vital pulp therapy (VPT), and regenerative endodontic procedures (REPs) have been proposed for treatment of dental pulp diseases. The purpose of VPT and REPs is to preserve pulp vitality and regenerate a new pulp-dentin complex [1]. Repair procedures are based on regulating cell fate, including their proliferation, migration, and differentiation [2]. Within the pulp, it has been shown that several cell populations participate in tissue regeneration [3]. Dental pulp stem cells (DPSCs) are isolated from the dental pulp of permanent teeth, the cells displaying multilineage differentiation capability, differentiating into odontoblast-like cells and undertaking dentin formation after stimulation [4]. Therefore, regulating the behavior of DPSCs is crucial for REPs and VPT.

Growth factors (GFs) are directly involved in modulating cellular events via the induction of specific intracellular signaling pathways [5–7]. Transforming growth factor-*β*1 (TGF-*β*1) is a ubiquitous multifunctional growth factor stored as a latent complex in the pulp's extracellular matrix (ECM) [8]. TGF-*β*1 can be activated following caries or pulpitis, subsequently affecting odontoblast-like cell differentiation and reparative dentinogenesis [9, 10]. TGF-*β*1 is also embedded within the dentin matrix [11] and can be released under specific local environmental conditions to direct cellular activity; for example, when bacterially derived lactic acid demineralizes dentin [12] or when the root canal is irrigated with ethylenediaminetetraacetic acid (EDTA) [13]. Exogenous application of TGF-*β*1 has been shown to elicit the same biological effect as its endogenous counterpart. Consequently, it can be combined with biomaterials to control cell fate during direct pulp capping or transplantation procedures within root canals [5, 14]. Indeed, the elucidation of how TGF-*β*1 regulates DPSCs differentiation has the potential to enable development of more targeted REPs and VPT.

Previous studies on the effect of TGF-*β*1 on dentinogenesis and mineralization have generated contrasting data. Reportedly, TGF-*β*1 can induce odontogenic differentiation of DPSCs and odontoblast-like cells *in vitro* [15, 16]. Indeed, it has been shown that capping pulp with a TGF-*β*1-containing material can induce odontoblast-like formation in dog molars [14]. However, a study using transgenic mice overexpressing TGF-*β*1 demonstrated defective mineralization in the tooth, potentially resulting from reduced dentin sialophosphoprotein (DSPP) levels [17]. Furthermore, TGF-*β*1 has been shown to downregulate odontogenic markers such as alkaline phosphatase (ALP), DSPP, and dentin matrix protein 1 (DMP-1) during odontoblast differentiation *in vitro* [18]. In addition, our previous reports have confirmed the inhibitory effect of TGF-*β*1 on stem cells differentiation from apical papilla (SCAP) and odontoblast-like cells [19, 20]. These discrepancies may be attributed to the treatment concentration, incubation time, cell type, and differentiation stage.

Given the known importance of TGF-*β*1 in dental tissue regeneration and the current lack of clarity relating to its role in dental cell responses, we investigated further how TGF-*β*1 affects odontogenic differentiation. Consequently, we observed a dual role mediated by TGF-*β*1 at different stages of DPSC differentiation.

## 2. Materials and Methods

### 2.1. Cell Isolation and Culture

Dental pulp tissues were isolated from healthy wisdom teeth of patients aged between 18 and 22 years at the Stomatological Hospital of the Air Force Medical University. All protocols were approved by the institutional review board. Briefly, the pulp tissue was minced into small pieces and dissociated with 4 mg/ml type I collagenase (Gibco, USA) at 37°C for 1 h. Furthermore, the suspension was filtered and transferred into a 6-well plate containing *α*-minimum essential medium (Gibco) supplemented with 20% fetal bovine serum (FBS, Gibco) and 1% penicillin–streptomycin (NCM Biotech, China). Cells were harvested with 0.25% (w/v) trypsin-EDTA (HyClone, USA) upon reaching 80% confluence. Single cell-derived colony cultures were obtained using the limiting dilution technique and subcultured in growth medium (*α*-MEM, 10%FBS and 1% penicillin–streptomycin) for further experiments. Three to five passages were used for this experiment.

For osteogenic differentiation, cells were treated with odonto-/osteo-genic medium (OM) (*α*-MEM, 10%FBS, 1% penicillin–streptomycin, 50 mg/mL ascorbic acid, 10 mM *β*-glycerophosphate, and 10 nM dexamethasone [Sigma-Aldrich, USA]) for 2 weeks and then were stained by Alizarin Red. For adipogenic differentiation, cells were cultured in adipogenic medium (*α*-MEM, 10% FBS, 1% penicillin-streptomycin, 0.1 *μ*M dexamethasone, 0.2 mM indomethacin, 0.01 mg/mL insulin, and 0.5 mM IBMX [Sigma-Aldrich]) for 4 weeks and stained with 0.5% Oil Red O and hematoxylin (Sigma-Aldrich). For chondrogenic differentiation, cells were centrifuged in a 15 mL polypropylene culture tube and cultured in freshly prepared chondrogenic medium (Cyagen, China) for 4 weeks. The chondrogenic pellets were fixed and paraffin embedded for Alcian blue staining (Cyagen).

The expression of stem cell associated phenotypic markers was analyzed by flow cytometry. Cells were incubated with specific antibodies for CD29, CD34, CD45, CD90, CD105, CD146, and isotype control (1 : 100; BioLegend, USA) for 1 h at room temperature and analyzed by Becton and Dickinson flow cytometry. The data were analyzed with the Mod-Fit 2.0 cell cycle analysis program (Becton and Dickinson).

### 2.2. Cell Counting Kit-8 (CCK-8) Assay

DPSCs were seeded at 3 × 10^3^ cells/well into 96-well plates containing medium supplemented with 2% fetal bovine serum and exposed to 0.1, 0.5, 1, and 5 ng/mL TGF-*β*1 (PeproTech, USA), with no exposure established as the negative control. Subsequently, 10 *μ*L of CCK-8 solution (Dojindo, Japan) was added to each well after 1, 3, 5, and 7 days of stimulation, and the plate was incubated at 37°C for 2 h. Optical density was measured at 450 nm using a microplate reader (Power Wave 340, Bio-TEK, USA).

### 2.3. Cell Cycle Analysis and Apoptosis Assay

Approximately 1 × 10^6^ cells were seeded into 60 mm^2^ culture dishes. After adherence, the cells were treated with the range of TGF-*β*1 concentrations, as described in Section 2.2, for 48 h. For cell cycle analysis, cells were collected and fixed by dropwise addition of 70% ethanol, followed by incubation with 500 *μ*L PI/RNase staining buffer (BD Pharmingen, USA) for 15 min. Apoptotic cells were quantified using the FITC Annexin V Apoptosis Detection Kit I (BD Pharmingen), as recommended by the manufacturer. All samples were assessed using flow cytometry (Beckman Coulter-XL, USA) and ModFit 3.0.

### 2.4. ALP Staining and ALP Activity

DPSCs were cultured in OM with or without TGF-*β*1 supplementation (range 0.1-5 ng/mL) for 7 days, or incubated in OM with or without 1 ng/mL TGF-*β*1 for 3, 5, 7, and 14 days. For ALP staining, cells were fixed with 4% paraformaldehyde, and an ALP staining kit (Beyotime, China) was used, following the manufacturer's instructions. ALP activity was analyzed using an ALP activity colorimetric assay kit (Beyotime) and determined by monitoring the absorbance at 405 nm using p-nitrophenyl phosphate as the substrate.

### 2.5. Alizarin Red S (ARS) Staining and Quantification

ARS staining was performed after 14 days of incubation in OM with the TGF-*β*1 concentrations ranging from 0.1-5 ng/mL or at day 5, 7, 14, and 21 after treatment with 1 ng/mL TGF-*β*1, no supplementation with TGF-*β*1 served as the negative control group. After fixing with 4% paraformaldehyde, samples were stained with 2% Alizarin Red S solution (pH 4.2; Sigma-Aldrich) for 10 min at room temperature. To determine the degree of mineralization, the cells and matrix were destained using 10% cetylpyridinium chloride for 30 min, and the absorbance of the solution was measured at 562 nm using a microplate reader (Power Wave 340).

### 2.6. Western Blot Analysis

Total protein was extracted using RIPA lysate with Protease Inhibitor Cocktail (Roche, Germany), and protein concentrations were measured using a bicinchoninic acid protein assay (Beyotime). Proteins (20 *μ*g) were separated using sodium dodecyl sulfate-polyacrylamide gel electrophoresis (Bio-Rad, USA) and transferred onto a polyvinylidene fluoride (PVDF) membrane (EMD Millipore, USA). After blocking in QuickBlock Blocking Buffer (Beyotime), the membrane was cut according to the molecular weight of the prestained marker protein and incubated overnight at 4°C with primary antibodies. Immune complexes were incubated with horseradish peroxidase-conjugated anti-rabbit or anti-mouse IgG antibodies at a concentration of 1 : 6000 (Yeasen, China). Enhanced chemiluminescence reagents (EMD Millipore) were added to visualize protein bands, and images were captured using a ChemiDoc MP system (Bio-Rad). ImageJ software (National Institutes of Health, Bethesda, MD) was used to calculate the intensity (gray value) of each protein band to enable comparison. Primary antibodies were purchased from the following commercial sources: collagen type I alpha 1 (COL1A1), bone sialoprotein (BSP) (1 : 1000; CST, USA), DSPP, osteocalcin (OCN) (1; 200; Santa Cruz Biotechnology, USA), DMP-1 (1 : 1000; Novus, USA), and glyceraldenhyde-3-phosphate dehydrogenase (GAPDH) (1 : 10000; Proteintech, USA).

### 2.7. Quantitative Real-Time Polymerase Chain Reaction (qRT-PCR) Assay

Total RNA was isolated from DPSCs using Trizol reagent (Invitrogen, USA) and reverse-transcribed to complementary DNA (cDNA) using a Reverse Transcription Reagent Kit (Applied Biological Materials, Canada) according to the manufacturer's instructions. qRT-PCR was performed using the CFX96 System (Bio-Rad, Berkeley, USA), and the BlasTaq 2X qPCR MasterMix (Applied Biological Materials) was used for qRT-PCR analysis. Data were normalized to GAPDH expression in each sample. Relative gene expression levels were calculated using ΔΔCt values. Primer sequences of target genes are detailed in Table 1.

### 2.8. *In Vivo* Odontogenic Assay

VitroGel 3D solution (TheWell Bioscience, USA) was used as a scaffold, as previously described [21]. All protocols were approved by the institutional review board. Briefly, cells (1 × 10^7^) were suspended in growth medium (GM) (*α*-MEM, 10%FBS, 1% penicillin–streptomycin), OM, or OM with TGF-*β*1 (final concentration of 1 ng/mL). The diluted hydrogel solution was gently mixed with cell suspensions, and the mixture was cultured in a 6-well plate. After five days, the mixtures were injected into subcutaneous pockets in the dorsal region of 6-week-old NOD/SCID mice (Air Force Medical University, China). After eight weeks, the mice were sacrificed by cervical dislocation under general anesthesia. Specimens were stained with Masson's trichrome staining (Servicebio, China), von Kossa staining (Servicebio), and immunofluorescence staining (Beyotime), according to the manufacturer's instructions and using previously reported protocols [21]. Human nuclear antigen antibodies were purchased from Abcam (1 : 100). Images were viewed and captured under an inverted microscope (Nikon, Japan) and using a confocal laser scanning microscope (A1 plus, Nikon, Japan).

### 2.9. Statistical Analyses

All experiments were performed at least in triplicate. Data are presented as mean ± standard deviation (SD). Comparisons between groups were performed using one-way or two-way ANOVA with GraphPad 8 (GraphPad Software, Inc., USA), and asterisks indicate significant differences (^∗^*p* < 0.05, ^∗∗^*p* < 0.01, ^∗∗∗^*p* < 0.001) compared with the control group.

## 3. Results

### 3.1. DPSC Identification

**Figure 1:**
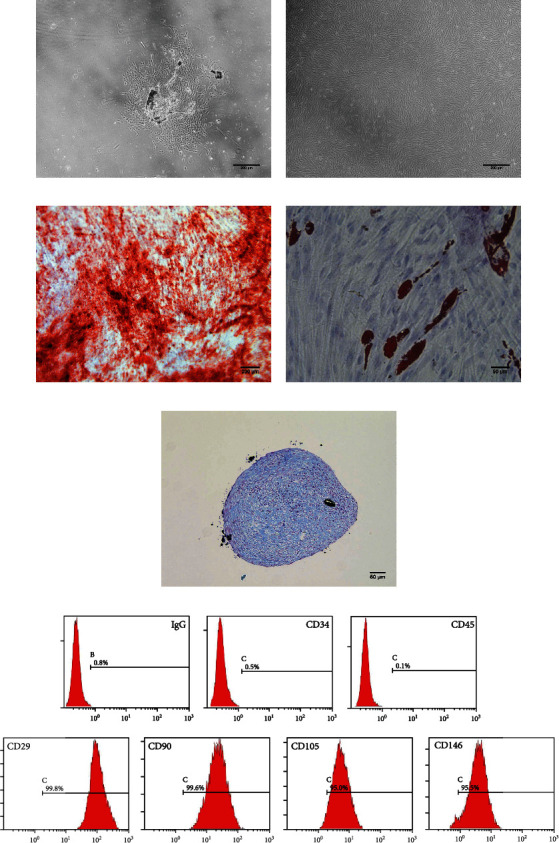
Characterization of DPSCs. (a) Primary DPSCs cultures on day 5 (40×). (b) DPSCs at passage 3 exhibit a spindle-shaped morphology (40×). (c) Mineralized nodules in DPSCs cultures stained with Alizarin Red S (50×). (d) Oil Red O staining for lipid droplets in DPSCs cultures (200×). (e) Alcian blue staining indicates synthesis of proteoglycans by chondrocytes (100×). (f) Molecular surface marker expression in DPSCs by flow cytometry. DPSCs: dental pulp stem cells. Scale bars are shown.

Clone-like growth of primary cells emerged after 5 days culture (Figure 1(a)). The cells obtained from clones appeared spindle-shaped (Figure 1(b)), forming mineralized nodules (Figure 1(c)), lipid droplets (Figure 1(d)) or chondrocytes (Figure 1(e)) under osteogenic, adipogenic, or chondrogenic medium induction. In addition, DPSCs exhibited high expression of mesenchymal surface markers (CD29, CD90, CD105, and CD146), while expression of hematopoietic markers (CD34 and CD45) was relatively low (Figure 1(f)).

### 3.2. TGF-*β*1-Mediated Inhibition of Cell Proliferation and Cell Cycle Arrest

**Figure 2:**
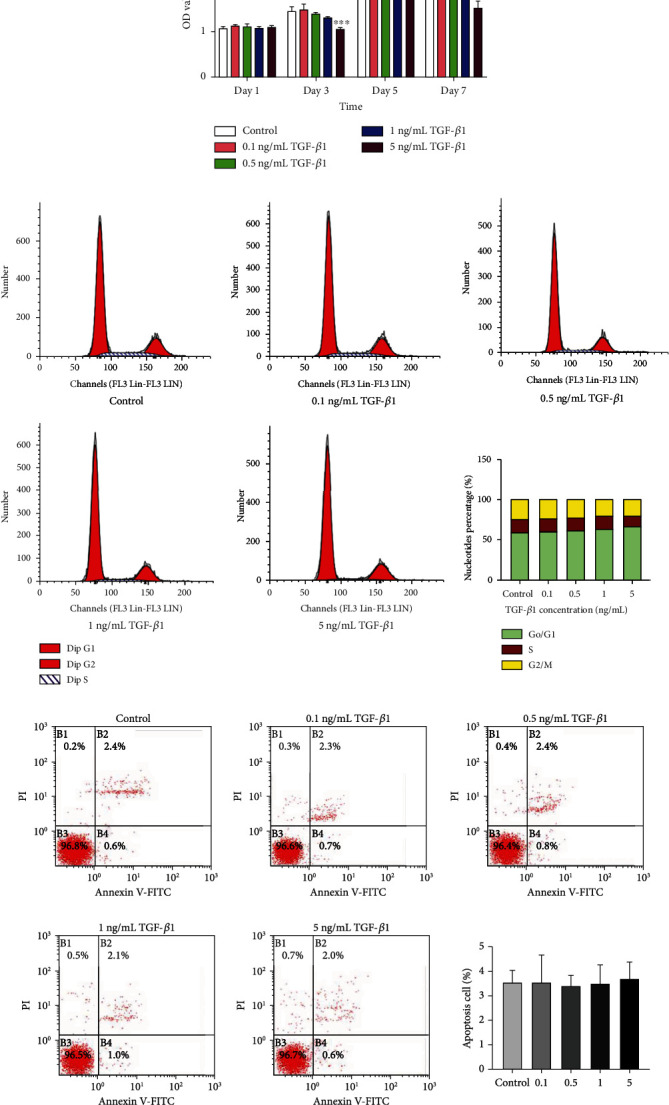
Effects of TGF-*β*1 on cell behavior (a) CCK-8 assay of DPSCs proliferation after treatment with a range of concentrations of TGF-*β*1 (0-5 ng/mL) for 1, 3, 5, and 7 days. (b) Flow cytometry analysis showing changes in the cell cycle of DPSCs following treatment with a range of concentrations of TGF-*β*1 for 48 h. (c) Cell apoptosis following stimulation with the TGF-*β*1 concentrations for 48 h evaluated by flow cytometry. Error bars = means ± standard deviation (SD), *n* = 3, statistically significant difference at the levels ^∗^*p* < 0.05, ^∗∗^*p* < 0.01, and ^∗∗∗^*p* < 0.001 (ANOVA) compared with the control group. CCK-8: cell counting kit-8; DPSCs: dental pulp stem cells; TGF-*β*1: transforming growth factor-*β*1.

Based on findings of the CCK-8 assay, 5 ng/mL TGF-*β*1 inhibited DPSC proliferation after 3 days of exposure, while treatment at the higher concentrations gradually inhibited cell proliferation at later stages (Figure 2(a)). After 48 h of stimulation, TGF-*β*1 exposure significantly increased the number of cells in the G1 phase and decreased those in the S phase in the experimental group dose-dependently (Figure 2(b)). No statistical differences in the percentage of early- and late-stage apoptotic cells were observed between exposure groups (Figure 2(c)).

### 3.3. TGF-*β*1 Promoted Early-Stage Differentiation but Inhibited the Mineralization Stage of DPSC Differentiation

**Figure 3:**
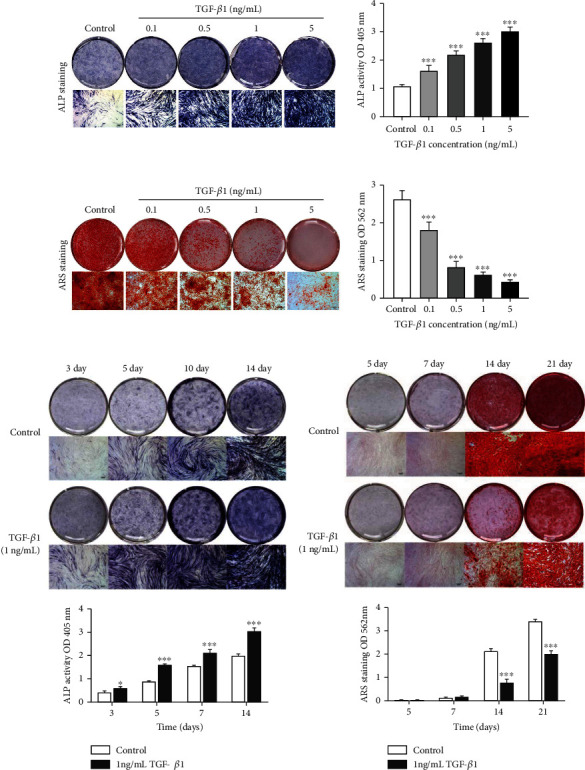
Effect of TGF-*β*1 on odontogenic differentiation of DPSCs *in vitro* (a) Images of ALP staining and ALP activity of DPSCs following range of concentrations of TGF-*β*1 (0-5 ng/mL) exposure for 7 days (50×). (b) Images and quantitative measurement of calcium mineral deposition stained by ARS staining in DPSCs cultured with the TGF-*β*1 concentrations for 14 days (50×). (c) Images of ALP staining and ALP activity of cells incubated with 1 ng/mL TGF-*β*1 at a range of time-points (days 3, 5, 10, 14) (50×). (d) Images and quantitative measurement of calcium mineral deposition stained by ARS staining in DPSCs following 1 ng/mL TGF-*β*1 exposure at different time-points (days 5, 7, 14, 21) (50×). Error bars = means ± standard deviation (SD), *n* = 3, statistically significant difference at the levels ^∗^*p* < 0.05 and ^∗∗∗^*p* < 0.001 (ANOVA) compared with the control group. ALP: alkaline phosphatase; ARS: Alizarin Red S; DPSCs: dental pulp stem cells; TGF-*β*1: transforming growth factor-*β*1.

ALP staining was more intense when DPSCs were cultured with TGF-*β*1, and this effect was dose-dependent at day 7. Increased ALP activities were consistent with elevated ALP staining levels in the cells with increasing concentrations of TGF-*β*1 (Figure 3(a)). In contrast, as shown in Figure 3(b), calcium deposition, as determined by ARS, was notably decreased in a dose-dependent manner. In addition, we found that continuous exposure to 1 ng/mL TGF-*β*1 resulted in the DPSCs exhibiting a gradual and time-dependent increase in ALP up to 14 days (matrix secretion and maturation stage) (Figure 3(c)). There were no differences between the two groups in ARS staining at days 5 and 7, but the intensity of ARS staining in groups treated with TGF-*β*1 was significantly decreased at days 14 and 21 (matrix mineralization stage) (Figure 3(d)).

**Figure 4:**
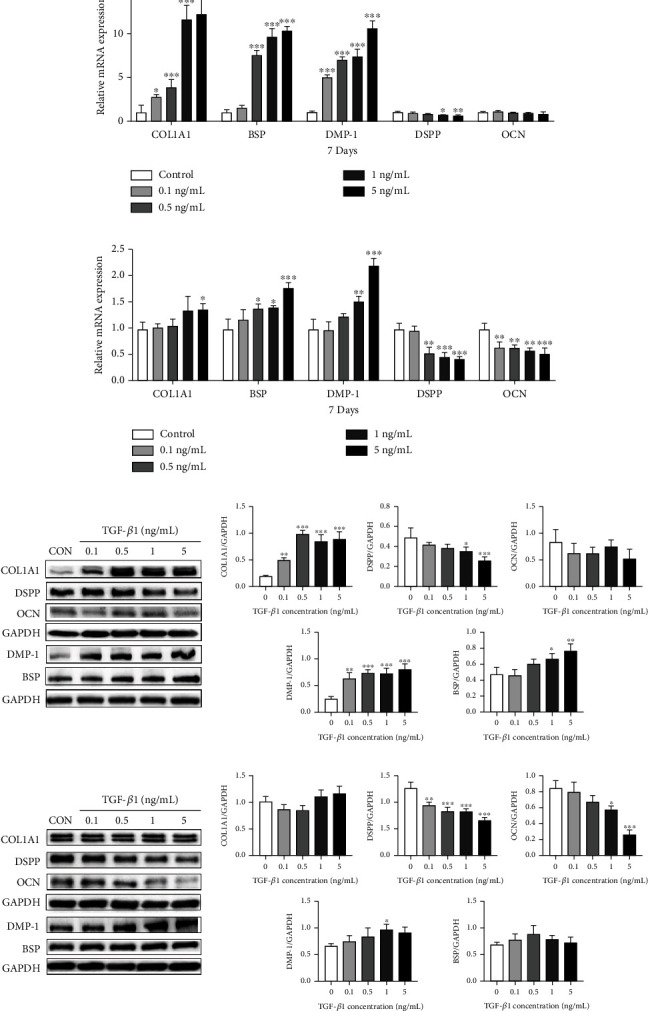
Effect of TGF-*β*1 on odonto-/osteo-genic-related marker gene and protein expression in DPSCs *in vitro* (a) The expression levels of COL1A1, DSPP, OCN, BSP, and DMP-1 were detected by qRT-PCR at day 7. (b) Relative gene expression levels of the genes at day 14. (c) Western blot analysis and quantification of proteins expression at day 7 after TGF-*β*1 exposure. (d) Analysis of protein expression after 14 days. Error bars = means ± standard deviation (SD), *n* = 3, statistically significant difference at the levels ^∗^*p* < 0.05, ^∗∗^*p* < 0.01, and ^∗∗∗^*p* < 0.001 (ANOVA) compared with the control group. BSP: bone sialoprotein; COL1A1: collagen type 1 alpha 1; DMP-1: dentin matrix protein 1; DPSCs: dental pulp stem cells; DSPP: dentin sialophosphoprotein; OCN: osteocalcin; TGF-*β*1: transforming growth factor-*β*1.

Furthermore, on day 7, qRT-PCR analysis showed that the range of concentrations TGF-*β*1 (0.1-5 ng/mL) stimulated upregulation of COL1A1 and DMP-1 mRNA expression as compared with untreated control. The levels of BSP mRNA were elevated in all treatment groups except the 0.1 ng/mL TGF-*β*1 exposure. The levels of DSPP mRNA were reduced in groups stimulated with the relatively high concentrations of TGF-*β*1 (1 and 5 ng/mL). No significant difference in OCN mRNA expression was observed between the exposure groups (Figure 4(a)). The protein expression levels of COL1A1, DMP-1, DSPP, and OCN showed a similar profile to that observed for the gene expression. BSP levels were increased in 1 and 5 ng/mL groups (Figure 4(c)). After incubation with TGF-*β*1 for 14 days, the variation of COL1A1, DMP-1, and BSP mRNA expressions was decreased between the groups, with a statistical difference only detected in the higher concentration groups. The levels of DSPP and OCN mRNA decreased in a concentration dependent manner (Figure 4(b)). The changes in protein expression in each group were not entirely the same observed for the mRNA expressions, however they appeared to follow a similar trend. Changes in COL1A1 and BSP expression were not statistically significant between groups, and DMP-1 expression levels were only marginally increased in the 1 ng/mL exposure group. DSPP expression decreased significantly following TGF-*β*1 stimulation in a dose-dependent manner, and the protein level of OCN decreased in groups stimulated with high TGF-*β*1 concentrations. (Figure 4(d)).

### 3.4. TGF-*β*1 Exposure Promoted Collagen Formation but Not Mineralization *In Vivo*

**Figure 5:**
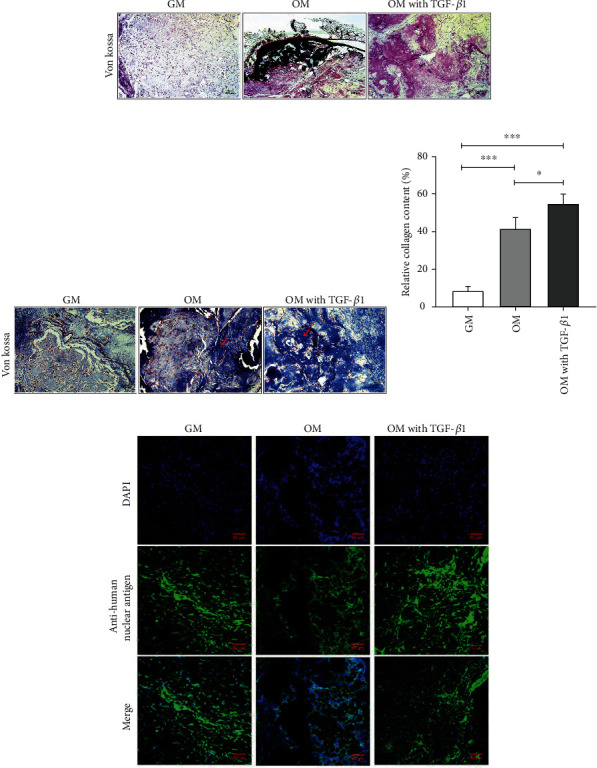
Effect of TGF-*β*1 on odontogenic differentiation of DPSCs *in vivo*. (a) Von Kossa staining in GM, OM, and TGF-*β*1 groups (blue arrow indicates hard tissue formation) (50×). (b) Collagen is dark blue following Masson trichrome staining (red arrows) (100×). (c) Measurement of the area of collagen formation. Error bars = means ± standard deviation (SD), *n* = 3, statistically significant difference at the levels ^∗^*p* < 0.05 and ^∗∗∗^*p* < 0.001 (ANOVA). (d) Indirect immunofluorescence for human nuclear antigen (green fluorescence). GM: growth medium; DPSCs: dental pulp stem cells; OM: odonto-/osteo-genic medium; TGF-*β*1: transforming growth factor-*β*1.

Results from von Kossa staining demonstrated the presence of calcified mineral deposits as represented by dark nodules, and these were clearly observed in OM group but not in the other exposure groups (Figure 5(a)). Collagen deposition was abundant in the TGF-*β*1 group, as indicated by Masson's trichrome staining. Most areas were stained light blue in the OM group, and minimal collagen deposition was detected in the GM group (Figure 5(b)). The total area of dark blue staining was analyzed using ImageJ (Figure 5(c)). Indirect immunofluorescence tests revealed that cells were positive for antihuman nuclear antigen (Figure 5(d)), which excluded cells originating from mice.

## 4. Discussion

Previous studies have demonstrated that TGF-*β*1 plays a role in regulating dentin-pulp tissue repair [22]. The biological effects of TGF-*β*1 on cells are contextual [23], depending on the culture medium, pretreatment procedures, incubation conditions, and more importantly, cell type and differentiation stage [24, 25]. Furthermore, studies have shown that TGF-*β*1 exerts different functionality during odontogenic differentiation [15, 19, 26]. Our study is now the first report to describe the dual role of TGF-*β*1 in DPSC differentiation, and now shows that TGF-*β*1 (at ˂5 ng/mL) facilitated early phases and modulated later phases of differentiation.

During tissue regeneration and repair, DPSCs differentiate into odontoblast-like cells to form dentin-like tissues. This odontoblast-like cell differentiation process is similar to that observed for osteoblast-mediated bone formation, which can be divided into three developmental stages of proliferation, early differentiation (matrix secretion and maturation), and late-stage differentiation (matrix mineralization) [27, 28]. Examining the different stages of differentiation can be routinely performed using histological staining techniques. Indeed, ALP levels are well characterized-indicator of early stages of differentiation, whereas later-stage mineralized nodule formation can be evaluated using ARS staining [29]. Previous studies assessing the effect of TGF-*β*1 on odontogenetic differentiation have shown that ALP activity was suppressed [18], and ALP and ARS analyses have demonstrated similar variations in cultured cells [16, 30]. These findings are in contrast with our data. In the present study, we detected increased ALP levels and decreased mineralization in groups treated with increasing TGF-*β*1 doses of up to 5 ng/mL. Consequently, we used hydrogels to continuously deliver GFs in scaffolds in animal studies [21, 31]. Compared with the OM group, the TGF-*β*1 treatment group formed abundant collagen, however no mineralized tissue was detected; these findings are consistent with the results observed *in vitro*. Collectively, we propose that TGF-*β*1 may stimulate matrix secretion and maturation, however sustained exposure impairs the mineralization processes in DPSCs. Notably, this is in agreement with similar studies performed in osteoblasts [32, 33], which have reported the dual and stage-dependent role of TGF*β*1 in the differentiation process and reported on the underlying mechanisms. Therefore, similar studies should be conducted to better understand the regulatory mechanisms exerted by TGF-*β*1 on DPSC differentiation and mineralization processes.

It is well established that cell proliferation and differentiation are closely associated [34]. Prior to secreting the predentin matrix, odontoblasts and odontoblast-like cells are withdrawn from the cell cycle [35]. Our results revealed that TGF-*β*1 inhibited cell proliferation by blocking the cell cycle in the G0/G1 phase, without inducing apoptosis in the early differentiation stage [36]. These results indicate that TGF-*β*1 promotes the initial differentiation of DPSCs through cell cycle arrest.

Various collagen and noncollagen proteins are systematically expressed during DPSC differentiation and exhibit distinct functions [37]. Therefore, we speculated that the biphasic effect of TGF-*β*1 was achieved by regulating mineral-associated protein expression, and this was verified by qRT-PCR and Western blot analysis. The dentin matrix is primarily composed of type I collagen, and COL1A1 expression is related to cell secretory activity [38]. BSP is one of the major proteins in mineralized tissue and acts as a nucleator of the initial apatite crystals [28, 39], and previous researches have described the effect of BSP on reparative dentinogenesis [40, 41]. Past studies have shown that the expression of DMP-1 increased at the early phase of odontoblast differentiation and declined when the odontoblasts reached maturity *in vitro* and *in vivo* [27, 42]. Therefore, these three proteins are considered early indicators of osteoblastic and odontogenic differentiation. According to qRT-PCR and Western blot assay, relatively high levels of these proteins were detected on day 7 of culture in the experimental exposure groups indicating that TGF-*β*1 induced matrix secretion and maturation. DSPP is a major noncollagenous protein found in dentin and can potently bind Ca^2+^ ions and induce mineral formation [43]. qRT-PCR and Western blot analysis showed a significant decrease in DSPP in TGF-*β*1-treated cells, suggesting that TGF-*β*1 could suppress dentin matrix mineralization. Decreased OCN expression on day 14 after TGF-*β*1 exposure also indicated that TGF-*β*1 impaired dentin matrix mineralization, as OCN is known to be an important marker of osteogenic differentiation and regulates calcium metabolism [28, 38]. These results support our conclusion that TGF-*β*1 promotes early differentiation and impairs later phase processes.

As discussed above, TGF-*β*1 regulates cellular events by activating specific signaling pathways. Unfortunately, the present study did not explore the mechanisms underlying the TGF-*β*1-mediated effects on odontogenic differentiation of DPSCs. Notably, few studies exploring this signaling have been reported. Previous findings have, however, suggested that activation of Smad and nonSmad signaling, such as PI3K/Akt, p38, MEK1/ERK, and Wnt/*β*-catenin signaling, participates in processing odontogenic differentiation regulated by TGF-*β*1 [19, 25, 32, 44, 45]. It therefore would appear apparent that the effects of TGF-*β*1 depend on the regulation of multiple signaling pathways [46]. The contrasting results of previous studies may be related to the stage-specific effect of TGF-*β*1 signaling on odontoblastic development. To mitigate the inhibitory effect on mineralization, it will be necessary to identify signaling pathways involved in late-stage differentiation.

Based on the findings of the dual role of TGF-*β*1 in this study, this molecule has the potential to be delivered and released both spatially and temporally during treatment for therapeutic benefit. For example, core/shell microspheres containing TGF-*β*1 could be applied during the early stage of dentin-pulp regeneration, and an additional growth factor could be subsequently released at a later stage [5]. In addition, it is important to understand the duration of action of the endogenous TGF-*β*1 released from the dentin matrix and pulp under different environmental conditions. This knowledge could be used for the modulation of the negative effects of this molecule on matrix mineralization by appropriate application of antagonists.

## 5. Conclusion

Our results demonstrate that TGF-*β*1 exerts an inhibitory effect on DPSC proliferation and causes cell cycle arrest. TGF-*β*1 also stimulated matrix formation and maturation while inhibiting matrix mineralization. These results suggest that the dual functions of TGF-*β*1 should be considered while utilizing TGF-*β*1 in REPs and VPTs. A thorough understanding of the stage- and dose-depend effects of TGF-*β*1 on DPSCs and associated underlying mechanisms could be harnessed to provide more targeted clinical interventions.

## Figures and Tables

**Table 1:** Primers sequence for real-time qRT-PCR.

Gene	Forward	Reverse
BSP	CACTGGAGCCAATGCAGAAGA	TGGTGGGGTTGTAGGTTCAAA
COL1A1	GAGGGCCAAGACGAAGACATC	GAGGGCCAAGACGAAGACATC
DSPP	GAGGTAACACCAGGCACT	GAGGTAACACCAGGCACT
DMP-1	ACTGTGGAGTGACACCAGAACACA	AGCTGCAAAGTTATCATGCAGATCC
OCN	GCCAGGCAGGTGCGAAGC	GTCAGCCAACTCGTCACAGTCC
GAPDH	GCACCGTCAAGGCTGAGAAC	TGGTGAAGACGCCAGTGGA

## Data Availability

The data that support the findings of this study are included within the article and available from the corresponding author upon reasonable request.

## References

[1] Murray P. E., Garcia-Godoy F., Hargreaves K. M. (2007). Regenerative endodontics: a review of current status and a call for action. *Journal of Endodontia*.

[2] da Rosa W. L. O., Piva E., da Silva A. F. (2018). Disclosing the physiology of pulp tissue for vital pulp therapy. *International Endodontic Journal*.

[3] Kim S. G., Zheng Y., Zhou J. (2013). Dentin and dental pulp regeneration by the patient's endogenous cells. *Endod Topics*.

[4] Gronthos S., Mankani M., Brahim J., Robey P. G., Shi S. (2000). Postnatal human dental pulp stem cells (DPSCs) in vitro and in vivo. *Proceedings of the National Academy of Sciences of the United States of America*.

[5] Kim S. G. (2017). Biological molecules for the regeneration of the pulp-dentin complex. *Dental Clinics of North America*.

[6] Smith A. J., Cooper P. R. (2017). Regenerative endodontics: burning questions. *Journal of Endodontia*.

[7] Hashemi-Beni B., Khoroushi M., Foroughi M. R., Karbasi S., Khademi A. A. (2017). Tissue engineering: dentin - pulp complex regeneration approaches (a review). *Tissue & Cell*.

[8] Baker S. M., Sugars R. V., Wendel M. (2009). TGF-beta/extracellular matrix interactions in dentin matrix: a role in regulating sequestration and protection of bioactivity. *Calcified Tissue International*.

[9] Piattelli A., Rubini C., Fioroni M., Tripodi D., Strocchi R. (2004). Transforming growth factor-beta 1 (TGF-beta 1) expression in normal healthy pulps and in those with irreversible pulpitis. *International Endodontic Journal*.

[10] Sloan A. J., Perry H., Matthews J. B., Smith A. J. (2000). Transforming growth factor-beta isoform expression in mature human healthy and carious molar teeth. *The Histochemical Journal*.

[11] Ivica A., Deari S., Patcas R., Weber F. E., Zehnder M. (2020). Transforming growth factor beta 1 distribution and content in the root dentin of young mature and immature human premolars. *Journal of Endodontia*.

[12] Dung S. Z., Gregory R. L., Li Y., Stookey G. K. (1995). Effect of lactic acid and proteolytic enzymes on the release of organic matrix components from human root dentin. *Caries Research*.

[13] Zeng Q., Nguyen S., Zhang H. (2016). Release of growth factors into root canal by irrigations in regenerative endodontics. *Journal of Endodontia*.

[14] Li F., Liu X., Zhao S., Wu H., Xu H. H. (2014). Porous chitosan bilayer membrane containing TGF-*β*_1_ loaded microspheres for pulp capping and reparative dentin formation in a dog model. *Dental Materials*.

[15] Jiang L., Ayre W. N., Melling G. E. (2020). Liposomes loaded with transforming growth factor *β*1 promote odontogenic differentiation of dental pulp stem cells. *Journal of Dentistry*.

[16] Li Y., Lu X., Sun X., Bai S., Li S., Shi J. (2011). Odontoblast-like cell differentiation and dentin formation induced with TGF-*β*1. *Archives of Oral Biology*.

[17] Thyagarajan T., Sreenath T., Cho A., Wright J. T., Kulkarni A. B. (2001). Reduced expression of dentin sialophosphoprotein is associated with dysplastic dentin in mice overexpressing transforming growth factor-*β*1 in teeth. *The Journal of Biological Chemistry*.

[18] Lin P. S., Chang M. C., Chan C. P. (2011). Transforming growth factor *β*1 down-regulates RUNX-2 and alkaline phosphatase activity of human dental pulp cells via ALK5/Smad2/3 signaling. *Oral Surgery, Oral Medicine, Oral Pathology, Oral Radiology, and Endodontics*.

[19] He W., Zhang J., Niu Z. (2014). Regulatory interplay between NFIC and TGF-*β*1 in apical papilla-derived stem cells. *Journal of Dental Research*.

[20] He W. X., Niu Z. Y., Zhao S. L., Jin W. L., Gao J., Smith A. J. (2004). TGF-*β* activated Smad signalling leads to a Smad3-mediated down-regulation of DSPP in an odontoblast cell line. *Archives of Oral Biology*.

[21] Xiao M., Qiu J., Kuang R., Zhang B., Wang W., Yu Q. (2019). Synergistic effects of stromal cell-derived factor-1*α* and bone morphogenetic protein-2 treatment on odontogenic differentiation of human stem cells from apical papilla cultured in the VitroGel 3D system. *Cell and Tissue Research*.

[22] Graham L., Cooper P. R., Cassidy N., Nor J. E., Sloan A. J., Smith A. J. (2006). The effect of calcium hydroxide on solubilisation of bio-active dentine matrix components. *Biomaterials*.

[23] Morikawa M., Derynck R., Miyazono K. (2016). TGF-*β* and the TGF-*β* family: context-dependent roles in cell and tissue physiology. *Cold Spring Harbor Perspectives in Biology*.

[24] Walenda G., Abnaof K., Joussen S. (2013). TGF-beta1 does not induce senescence of multipotent mesenchymal stromal cells and has similar effects in early and late passages. *PLoS One*.

[25] Zhang Y., Zhang H., Yuan G., Yang G. (2021). Effects of transforming growth factor-*β*1 on odontoblastic differentiation in dental papilla cells is determined by IPO7 expression level. *Biochemical and Biophysical Research Communications*.

[26] Hwang Y. C., Hwang I. N., Oh W. M., Park J. C., Lee D. S., Son H. H. (2008). Influence of TGF-beta1 on the expression of BSP, DSP, TGF-beta1 receptor I and Smad proteins during reparative dentinogenesis. *Journal of Molecular Histology*.

[27] Lee D. S., Yoon W. J., Cho E. S. (2011). Crosstalk between nuclear factor I-C and transforming growth factor-*β*1 signaling regulates odontoblast differentiation and homeostasis. *PLoS One*.

[28] Amarasekara D. S., Kim S., Rho J. (2021). Regulation of osteoblast differentiation by cytokine networks. *International Journal of Molecular Sciences*.

[29] Macri-Pellizzeri L., De Melo N., Ahmed I., Grant D., Scammell B., Sottile V. (2018). Live quantitative monitoring of mineral deposition in stem cells using tetracycline hydrochloride. *Tissue Engineering. Part C, Methods*.

[30] Zhang J., Zhang C. F., Li Q. L., Chu C. H. (2019). Cyclic adenosine monophosphate promotes odonto/osteogenic differentiation of stem cells from the apical papilla via suppression of transforming growth factor Beta 1 signaling. *Journal of Endodontia*.

[31] Haugen H. J., Basu P., Sukul M., Mano J. F., Reseland J. E. (2020). Injectable biomaterials for dental tissue regeneration. *International Journal of Molecular Sciences*.

[32] Nam B., Park H., Lee Y. L. (2020). TGF*β*1 suppressed matrix mineralization of osteoblasts differentiation by regulating SMURF1–C/EBP*β*–DKK1 axis. *International Journal of Molecular Sciences*.

[33] Maeda S., Hayashi M., Komiya S., Imamura T., Miyazono K. (2004). Endogenous TGF-beta signaling suppresses maturation of osteoblastic mesenchymal cells. *The EMBO Journal*.

[34] Qin L., Li X., Ko J. K., Partridge N. C. (2005). Parathyroid hormone uses multiple mechanisms to arrest the cell cycle progression of osteoblastic cells from G_1_ to S phase. *The Journal of Biological Chemistry*.

[35] Almushayt A., Narayanan K., Zaki A. E., George A. (2006). Dentin matrix protein 1 induces cytodifferentiation of dental pulp stem cells into odontoblasts. *Gene Therapy*.

[36] Breen E. C., Ignotz R. A., McCabe L., Stein J. L., Stein G. S., Lian J. B. (1994). TGF*β* alters growth and differentiation related gene expression in proliferating osteoblasts in vitro, preventing development of the mature bone phenotype. *Journal of Cellular Physiology*.

[37] Goldberg M., Kulkarni A. B., Young M., Boskey A. (2011). Dentin: structure, composition and mineralization. *Frontiers in Bioscience (Elite Edition)*.

[38] Ching H. S., Luddin N., Rahman I. A., Ponnuraj K. T. (2017). Expression of odontogenic and osteogenic markers in DPSCs and SHED: a review. *Current Stem Cell Research & Therapy*.

[39] Qin C., Baba O., Butler W. T. (2004). Post-translationalmodifications ofSIBLING proteins andtheirroles inosteogenesis anddentinogenesis. *Critical Reviews in Oral Biology and Medicine*.

[40] Vijaykumar A., Dyrkacz P., Vidovic-Zdrilic I., Maye P., Mina M. (2020). Expression of BSP-GFPtpz transgene during osteogenesis and reparative dentinogenesis. *Journal of Dental Research*.

[41] Decup F., Six N., Palmier B. (2000). Bone sialoprotein-induced reparative dentinogenesis in the pulp of rat's molar. *Clinical Oral Investigations*.

[42] Hao J., Zou B., Narayanan K., George A. (2004). Differential expression patterns of the dentin matrix proteins during mineralized tissue formation. *Bone*.

[43] Linde A. (1995). Dentin mineralization and the role of odontoblasts in calcium transport. *Connective Tissue Research*.

[44] Chang H. H., Chang M. C., Wu I. H. (2015). Role of ALK5/Smad2/3 and MEK1/ERK signaling in transforming growth factor beta 1-modulated growth, collagen turnover, and differentiation of stem cells from apical papilla of human tooth. *Journal of Endodontia*.

[45] Shao M. Y., Cheng R., Wang F. M., Yang H., Cheng L., Hu T. (2011). *β*-Catenin and Rho GTPases as downstream targets of TGF-*β*1 during pulp repair. *Cell Biology International*.

[46] Grafe I., Alexander S., Peterson J. R. (2018). TGF-*β* family signaling in mesenchymal differentiation. *Cold Spring Harbor Perspectives in Biology*.

